# Light-Boosting Highly Sensitive and Ultrafast Piezoelectric Sensor Based on Composite Membrane of Copper Phthalocyanine and Graphene Oxide

**DOI:** 10.3390/ijms25126713

**Published:** 2024-06-18

**Authors:** Jihong Wang, Zhening Fang, Wenhao Liu, Liuyuan Zhu, Qiubo Pan, Zhen Gu, Huifeng Wang, Yingying Huang, Haiping Fang

**Affiliations:** 1 School of Physics, East China University of Science and Technology, Shanghai 200237, Chinahuangyingying@ecust.edu.cn (Y.H.); 2 School of Materials Science and Engineering, East China University of Science and Technology, Shanghai 200237, China; 3 Center for Transformative Science, ShanghaiTech University, Shanghai 200237, China; 4 Key Laboratory of Smart Manufacturing in Energy Chemical Process Ministry of Education, East China University of Science and Technology, Shanghai 200237, China; whuifeng@ecust.edu.cn

**Keywords:** piezoelectric sensor, light illumination, high sensitivity, ultrafast response

## Abstract

Self-powered wearable pressure sensors based on flexible electronics have emerged as a new trend due to the increasing demand for intelligent and portable devices. Improvements in pressure-sensing performance, including in the output voltage, sensitivity and response time, can greatly expand their related applications; however, this remains challenging. Here, we report on a highly sensitive piezoelectric sensor with novel light-boosting pressure-sensing performance, based on a composite membrane of copper phthalocyanine (CuPC) and graphene oxide (GO) (CuPC@GO). Under light illumination, the CuPC@GO piezoelectric sensor demonstrates a remarkable increase in output voltage (381.17 mV, 50 kPa) and sensitivity (116.80 mV/kPa, <5 kPa), which are approximately twice and three times of that the sensor without light illumination, respectively. Furthermore, light exposure significantly improves the response speed of the sensor with a response time of 38.04 µs and recovery time of 58.48 µs, while maintaining excellent mechanical stability even after 2000 cycles. Density functional theory calculations reveal that increased electron transfer from graphene to CuPC can occur when the CuPC is in the excited state, which indicates that the light illumination promotes the electron excitation of CuPC, and thus brings about the high polarization of the sensor. Importantly, these sensors exhibit universal spatial non-contact adjustability, highlighting their versatility and applicability in various settings.

## 1. Introduction

Pressure sensors are highly desired in various fields, e.g., in industrial production, medical equipment, automotive industry, aerospace, environmental monitoring, and food processing [1,2,3,4]. With the development of artificial intelligence, self-powered wearable devices based on flexible electronics are gradually becoming the mainstream of next-generation portable devices [5,6,7]. Undoubtedly, the piezoelectric sensor has emerged as a major research focus, necessitating continuous advancements to meet the diverse demands for large output voltage, high flexibility, rapid response, heightened sensitivity, and cost-effectiveness. Up to date, a significant number of studies have focused on carefully-designed microstructures of the pressure sensor to improve piezoelectric performance, but there are few reports investigating improvements in the pressure sensing performance of the sensor material itself under external conditions such as light illumination.

Light energy, as a renewable and widely accessible resource, offers the advantages of environmental sustainability, versatility, and cost-effectiveness. Therefore, harnessing light to regulate the piezoelectric effect presents a promising opportunity to not only further enhance the performance of pressure sensors but also extend the scope of optical control functionality. While the influence of light on piezoresistive pressure sensors has been previously reported [8], the complex preparation process and reliance on external power sources pose significant challenges. Notably, there have been no reports to date on the impact of light on the performance and mechanism of self-powered piezoelectric pressure sensors. The selection and preparation of sensor layer is very critical. Graphene oxide (GO), a derivative of graphene, possesses a two-dimensional network structure akin to graphene, along with carboxyl, hydroxyl, epoxy, and other oxygen-containing functional groups [9,10,11]. This unique structure endows GO with the advantageous qualities of graphene, including exceptional shielding properties, a high aspect ratio, extraordinary strength, remarkably high thermal conductivity, and substantial surface activity. However, the insulation, limited light absorption, and defect properties of GO restrict its potential applications in optics and electronics [12,13,14]. Fortunately, the presence of diverse oxygen-containing functional groups on the substrate and sheet edges enables GO to interact with a photoexcitable material, such as copper phthalocyanine (CuPC), in either a non-covalent or covalent doping manner, leading to the formation of hybrid structures and composites exhibiting extraordinary properties [15,16,17]. Both GO and organic molecules can be modeled to determine their electron affinity and spectral properties, facilitating the design of donor–acceptor pairs or charge–transfer complexes. Hybrid GO–photoexcitable materials offer a synergistic combination of exceptional carrier mobility and outstanding photoelectric properties, making them highly promising candidates for flexible piezoelectric devices that can be regulated by light [18,19,20].

In this study, we propose a novel approach that utilizes external light illumination to greatly enhance the piezoelectric performance of the sensor, which is based on a composite membrane of the traditional photoelectric material (CuPC) and graphene oxide (GO) (CuPC@GO). Under the condition of light illumination, the output voltage is almost doubled, reaching 381.17 mV under pressure of 50 kPa; the pressure-sensing sensitivity of the sensor is enhanced by two times, resulting in a high sensitivity of 116.80 mV/kPa below the pressure of 5 kPa; and the response time and recovery time are significantly improved to less than one-tenth, the corresponding values being 38.04 µs and 58.48 µs, respectively. Moreover, the sensor exhibits excellent mechanical stability, still maintaining 95% of its voltage output after 2000 cyclic loading and unloading experiments. This flexible and self-powered piezoelectric sensor can be non-contact and highly adjustable, representing a significant advancement in the field of continuous health monitoring and intelligent electronic devices.

## 2. Results and Discussion

### 2.1. Preparation of the CuPC@GO-Based Piezoelectric Sensor and Its Properties

**Figure 1:**
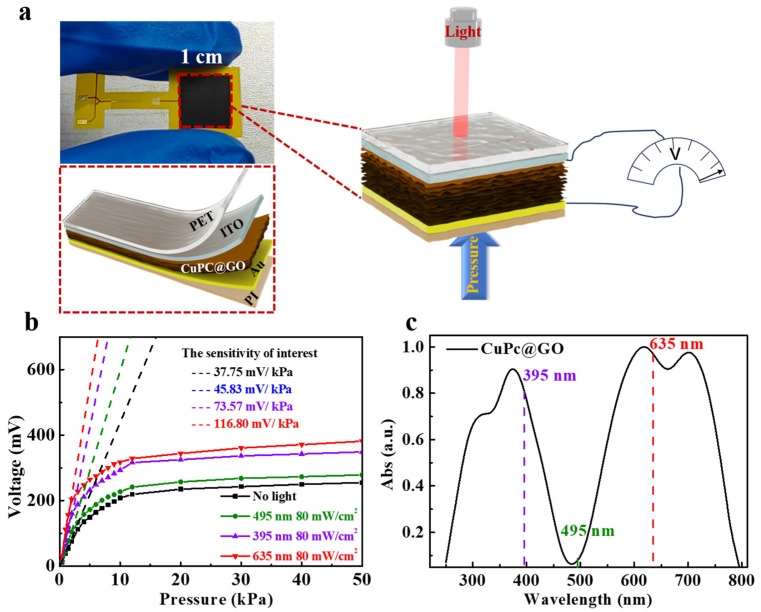
Schematic diagram of the CuPC@GO piezoelectric sensor and its performance under light illumination: (**a**) a brief schematic of the pressure response/light-boosting pressure response of flexible piezoelectric sensor based on the @GO membrane between conductive (PET) ITO/Au (PI) films, where pressure is applied on the PI film side and light is applied on the PET side; (**b**) voltage output signals of the CuPC@GO piezoelectric sensor at different wavelengths of 80 mW/cm^2^ optical power, and the unilluminated voltage output is taken as a control; and (**c**) ultraviolet–visible light absorption spectra of CuPC@GO composite films.

The CuPc@GO membrane was fabricated using the drip film method (see Methods), which served as the pressure-sensing layer. Subsequently, a CuPc@GO piezoelectric sensor with a polyethylene terephthalate (PET)/indium tin oxide (ITO)/CuPc@GO/Au/polyimide (PI) structure was obtained by utilizing ITO and Au as the two active electrodes (Figure 1a) and PET and PI as encapsulation layers. Under external mechanical stimulation, the close contact of the two electrodes with the sensing layer in piezoelectric sensors generates an electric potential difference, thus the electrical signal is produced between the electrodes in response to the external stimulus.

The generated electric potential was tested under different pressures, as depicted in Figure 1b. To comprehensively elucidate the influence of light illumination on piezoelectric properties, monochromatic light at 635 nm, 395 nm, and 495 nm with 80 mW/cm^2^ optical power were applied to the sensor, respectively, while the unilluminated condition served as the control group. Under light illumination, the voltage response of the piezoelectric sensor exhibited a significant enhancement across a wide range of applied pressures from 0 to 50 kPa. Notably, at a pressure of 50 kPa, the output voltage increased to 381.17 mV, demonstrating an increase of one time compared to the output voltage obtained without light illumination.

The relationship between pressure and voltage variation can be described by sensitivity, which is a crucial parameter of pressure sensors. The sensitivity (S) can be calculated by Equation (1) [21], as follows:

(1) $$S=\frac{\Delta V}{\Delta P}$$

where ΔV represents the change in the output voltage signal, and ΔP represents the change in the applied pressure (P). It can be inferred that the voltage-to-pressure curve exhibits a consistent trend irrespective of light illumination, while the voltage enhancement effect is the most pronounced within the pressure range of <5 kPa. Particularly, under the irradiation of 80 mW/cm^2^ optical power at a wavelength of 635 nm, the sensitivity of the sensor is 116.80 mV/kPa within *P* < 5 kPa. As a comparison, the sensitivity decreases to 37.75 mV/kPa without illumination, which represents that the sensitivity was increased by approximately three times under the external light illumination. The increase in sensitivity varies for different light wavelengths under the same light power. Under 635 and 395 nm illumination, the sensitivity increase is larger than that under 495 nm illumination.

The enhancement of the device sensitivity depends on the light absorption capacity of the CuPc@GO film. Herein, the UV–visible light absorption spectra of CuPc@GO films were examined, as shown in Figure 1b. The absorption intensity presents peaks as the incident light wavelength reached 635 and 395 nm, while a dip was observed for 495 nm. It was observed that the piezoelectric enhancement effect of different light wavelengths on the device aligns with the law of absorption intensity.

**Figure 2:**
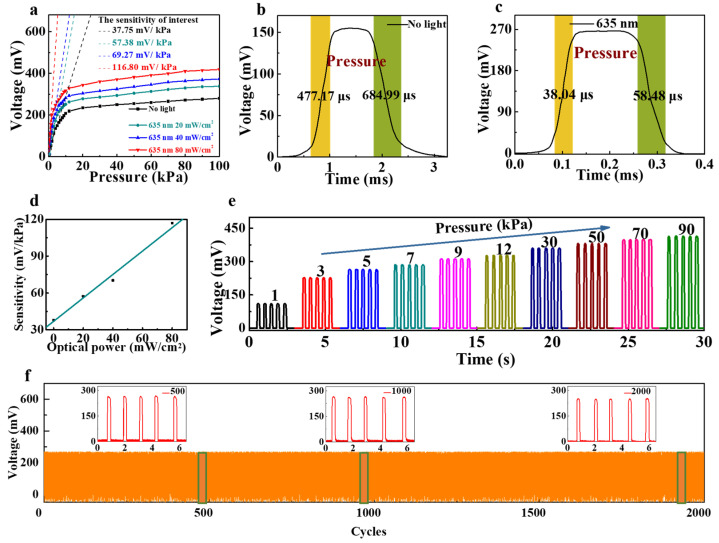
Pressure-sensing performance of the sensor: (**a**) voltage output signal of the sensor in the range of 0~100 kPa under different optical power of 635 nm light, and the voltage output without light is used as a control; (**b**,**c**) response/recovery time curves of the sensor with 635 nm light illumination and without light illumination; (**d**) sensitivity of the sensor under *P* < 5 kPa varying with optical power of 635 nm light; (**e**) output voltages of the sensor under different pressures; and (**f**) long-term cyclic stability of the sensor. The green squares represent the magnified areas around 500 cycles, 1000 cycles and 2000 cycles, respectively.

The generated electric potential also differs with the optical power of the external light illumination. As the light power of the 635 nm incident wave increases from 0 to 80 mW/cm^2^, the output voltage increases correspondingly (Figure 2a), and the corresponding sensitivity also increases linearly (Figure 2d), illustrating that the sensitivity of the sensor can be adjusted based on the varying optical power.

Response time, as a vital parameter in sensor performance evaluation, was also investigated. Here, we conducted an analysis of voltage signal variation curves over time under 80 mW/cm^2^ power illumination at a wavelength of 635 nm for comparison with that without light illumination (Figure 2b,c). Remarkably, when exposed to light and subjected to a pressure of 5 kPa, the response time achieved an impressive value of 38.04 µs, exhibiting an order of magnitude improvement compared to the response time of 477.17 µs obtained without light. Simultaneously, the recovery time of the piezoelectric sensor under light illumination decreased to 58.48 µs, exhibiting a substantial reduction compared to that (684.99 µs) in the absence of light illumination. Our findings demonstrate that the light illumination can significantly enhance the performance of the sensor.

Loading and unloading experiments were performed to examine the mechanical stability of the sensor. A periodic and step-like response of the output voltage can be clearly seen, which is well correlated with the applied pressure (Figure 2e). The output voltage remains stable when the external pressure remains constant and shows positive correlation with pressure as the external light is kept constant. To verify the stability and durability of the sensor during long-term operation, the loading and unloading analysis was then repeated for 2000 cycles at 5 kPa under the condition of 80 mw/cm^2^ power at a wavelength of 635 nm. As depicted in Figure 2f, the pressure response remained relatively stable throughout the cycle experiment, with an ultra-low potential consumption of less than 5% after 2000 cycles, ensuring the performance stability with over 95% output voltage.

### 2.2. Characterization of CuPC@GO Membrane as Pressure-Sensing Layer

**Figure 3:**
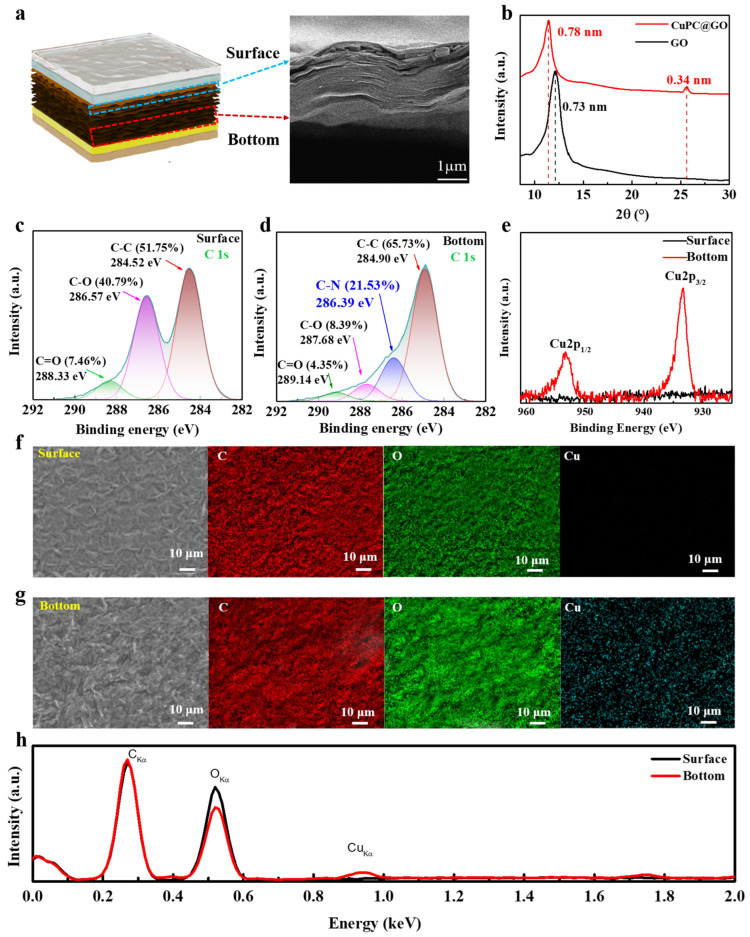
Characterization of CuPC@GO membrane: (**a**) scanning electron microscope (SEM) image of CuPC@GO membrane section; (**b**) X-ray diffraction (XRD) patterns of GO membrane and CuPC@GO membrane; (**c**,**d**) high-resolution C1s X-ray photoelectron spectroscopy (XPS) spectra of the surface and bottom parts of the CuPC@GO membrane; (**e**) high-resolution Cu 2p XPS spectra of the surface and bottom parts of the CuPC@GO membrane. SEM images and energy dispersive spectrometer (EDS) mapping of the (**f**) surface and (**g**) bottom parts of the CuPC@GO membrane; the elements of C, O and Cu are represented by red, green and fluorescent blue, respectively; and (**h**) EDS spectral analysis of the surface and bottom parts of the CuPC@GO membrane.

CuPC has gained significant attention for its remarkable photoelectric properties [22], but the easy aggregation during the formation process of related film hinders its wider application. Here, the CuPC@GO membrane was obtained by uniformly mixing GO dispersion with CuPC molecules and subsequently depositing the film, because the hydrophilic oxygen groups on GO enable them to easily disperse in water [23]. The cross-sectional scanning electron microscope (SEM) image (Figure 3a) shows that the CuPC@GO membrane is a well-organized layered structure by self-assembly. The X-ray diffraction (XRD) patterns of CuPC@GO membranes and GO membranes are further demonstrated in Figure 3b. The curves related to GO display intense and sharp peaks centered at 2θ = 12.12°, representing a (0 0 1) plane spacing of 0.73 nm. For the CuPC@GO membrane, the (0 0 1) peak is observed at 2θ = 11.38° (0.78 nm), indicating a slightly widened (0 0 1) plane spacing, as the CuPC molecules are inserted between the GO layers. Furthermore, compared to GO, the XRD pattern of the CuPC@GO membrane shows a novel peak at 2θ = 25.5°, indicating an interlayer distance of 0.34 nm. The new peak suggests that the CuPC was effectively combined with GO, obtaining a collective effect reflected as the extra interlayer distance.

To elucidate the microstructure of the CuPC@GO membrane in detail, X-ray photoelectron spectroscopy (XPS) was employed. The C1s spectrum of the upper surface of the CuPC@GO membrane and that of GO contain the same carbon (C) species, albeit in slightly different proportions (Figure 3c). However, the C species in the bottom layer of the CuPC@GO membrane exhibit a higher concentration of C-N bonds compared to those on the upper surface, coupled with a notable reduction in the proportion of C–O bonds (Figure 3d). Energy dispersive spectrometer (EDS) mapping and EDS spectral analysis in Figure 3f–h show that the upper surface of the CuPC@GO membrane solely comprises GO, while the bottom layers consist of CuPC–GO complexes.

To further explore the nature of the bonding between GO and CuPC, the Cu 2p spectrum was analyzed (Figure 3e). At the upper surface of the CuPC@GO membrane, no peaks corresponding to the Cu 2p spectral signature are observed. However, two strong peaks appear in the Cu 2p spectrum are acquired from the bottom layer of the CuPC@GO membrane, namely, at ~953.3 eV and ~933.3 eV, which can be attributed to the electronic states of Cu 2p_1/2_ and Cu 2p_3/2_, respectively. It is noteworthy that the binding energy of the Cu 2p_3/2_ electron state of the copper atom corresponds to the oxidation state of Cu (932.8 eV) in the CuPC molecule; however, the Cu 2p_3/2_ signal is altered by 0.5 eV. This phenomenon can be attributed to the formation of metal–ligand coordination bonds between the oxygen functional groups of GO and the central metal ion of CuPC in the deep layer of the CuPC@GO membrane. The structure of the CuPC@GO membrane can be understood as a consequence of the competition between metal–ligand coordination and π-π interactions between GO and CuPC. These results are consistent with the XPS results in that the upper surface of the membrane contains no CuPC component, and strong asymmetry by its self-assembly is observed, which is essential for its piezoelectric performance.

### 2.3. Physical Mechanism of Light-Boosting Pressure-Sensing Performance of the Sensor

**Figure 4:**
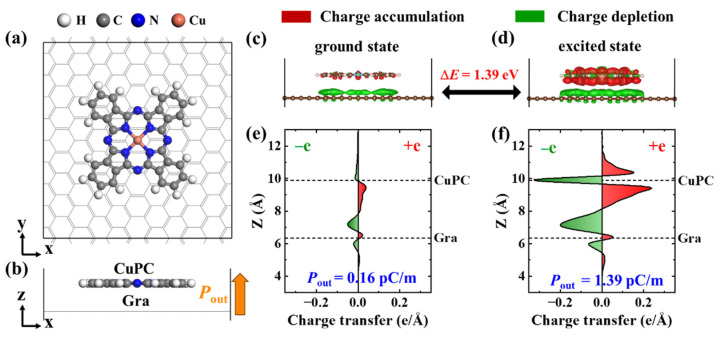
Physical mechanism of light-boosting sensing performance of the sensor: (**a**,**b**) top view and side view of the structure for CuPC on graphene surface (CuPC@Gra). The white, grey, blue and orange balls represent hydrogen, carbon, nitrogen and cupper atoms, respectively; (**c**,**d**) differential charge density plots of ground state and excited state of CuPC@Gra, with an iso-surface value of 1.5 × 10^−4^ e/bohr^3^; and (**e**,**f**) plane-averaged differential charge density in z direction for ground state and excited state of CuPC@Gra, respectively. Red denotes the charge accumulation and green denotes the charge depletion.

We performed density functional theory (DFT) calculations to illustrate the mechanism of the light-boosting sensing performance of the sensor. As shown in Figure 4a,b, the structure of CuPC on a graphene surface (CuPC@Gra) was constructed. The ground state and the excited state of CuPC@Gra were considered based on the condition of light illumination. The energy difference between the ground state and the excited state of CuPC@Gra is 1.39 eV, which corresponds to the energy required for an electron transition. This electron emission energy roughly corresponds to the photoexcitation energy (1.90 eV) of the light wavelength of 635 nm. The differential charge densities (DCDs) in the *z* direction for the ground state and the excited state of CuPC@Gra were analyzed (Figure 4c–f). The electron was transferred from graphene to CuPC, thus leading to a polarization direction pointing from graphene to CuPC. Compared with the ground state of CuPC@Gra, the polarization (P_out_) of the excited state of CuPC@Gra was increased by one order of magnitude (1.39 pC/m), which indicates that the light illumination can promote more electron transference from graphene to CuPC. Further, the ground state and the excited state of one CuPC were calculated; the energy difference between them was shown to be 1.28 eV, which roughly corresponds to the photoexcitation energy of the light wavelength of 635 nm. These results indicate that the light illumination can promote the electron emission of CuPC molecule, which meant that more electrons were transferred from CuPC to graphene, further resulting in the large polarization and the enhancement of the piezoelectric performance of the sensor.

### 2.4. Application of Multimodal Sensing Neuromorphic Sensor

In this section, we demonstrate one of the applications of ultra-high piezoelectric response through light illumination to neuromorphic sensors. Traditional audio-based classification systems, constrained by the single-dimensional signal input of detectors, are limited to analyzing signals along the temporal dimension, resulting in suboptimal accuracy. Conversely, multi-dimensional perceptual systems necessitate various functional detectors and intricate neural network architectures. Utilizing the CuPC@GO piezoelectric sensor to enhance the piezoelectric response through illumination, detectors can not only capture pure audio signals but also discern the morphology of the target object through the amplitude enhancement of audio signals at different detector positions. 

**Figure 5:**
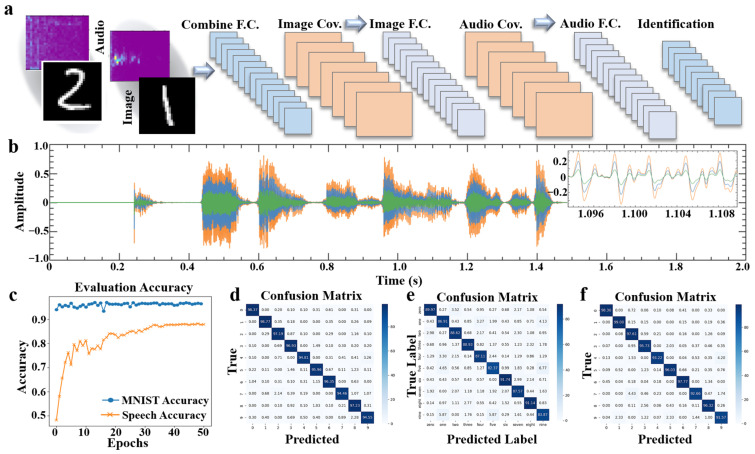
Application of multimodal sensing neuromorphic sensor: (**a**) the layered structure of the multimodal neural network; (**b**) detector response to audio input over time under varying optical power. The orange curve represents the detector unit’s response under high power illumination, while the blue and green curves correspond to the detector unit’s responses under medium and low power illumination conditions, respectively; (**c**) the variation in tested accuracy with training epochs for the MNIST dataset and the Speech Command dataset; and (**d**–**f**) the confusion matrices for the MNIST dataset, the Speech Command dataset, and the combined network.

We fabricated a CuPC@GO detector array and devised a multimodal perception neural network based on this detector. To demonstrate the application value of the material and the neural network model construction, the architecture of the neural network model is depicted in Figure 5a. Audio signals ranging from 0 to 9 in the Speech Command dataset were inputted into convolutional neural networks (CNNs) with two convolutional layers of 50 output channels and 7 × 7 dimension feature maps, followed by one fully connected layer. The resulting accuracy is 87.88%, detailed in Figure 5c. As a point of comparison, alongside the input of audio signals, images depicting the distribution of ambient light are introduced. Different light intensities at various positions exert varying effects on the audio signals, which are reflected in the corresponding detector array, as illustrated in Figure 5b. Different color curves describe the relationship between the variation of light intensity and the corresponding audio signal enhancement. For the image-only input network, the MNIST dataset [24] was applied. The input data underwent two convolutional layers of four 7 × 7 feature maps and one fully connected layer; the resulting testing accuracy of 50 epochs is 96.49%, as shown in Figure 5c. The confusion matrix of this network is shown in Figure 5d. It is evident that employing multidimensional inputs significantly enhances accuracy. As shown in Figure 5e, the accuracy of each class of audio network is enhanced by the combine network; easy-to-confuse numbers like “five” and “nine”, with accuracies of 82.37% and 83.87%, were enhanced to 96.30% and 91.57% in the combined network, as shown in Figure 5f.

Thus, the CuPC@GO piezoelectric sensor, influenced by light illumination, holds extensive potential for various applications. By fully leveraging the acoustic and spatial characteristics of piezoelectric detectors, along with online training and analysis of light-induced pressure response curves, we offer a novel solution for the realization of complex multimodal neural networks, facilitating high-accuracy and low-power consumption classification and identification systems.

## 3. Methods

### 3.1. Preparation of the CuPC@GO Composite Membrane and the CuPC@GO Piezoelectric Pressure Sensor

GO were synthesized from natural graphite powder using a modified Hummers method [25,26,27]. The graphite powders were combined with concentrated H_2_SO_4_, K_2_S_2_O_8_, and P_2_O_5_ solution and stirred continuously for several hours. The resulting mixture was then diluted with ultrapure water, centrifuged, and washed with ultrapure water. Once dry, the preoxidized graphite was obtained and subsequently oxidized further in concentrated H_2_SO_4_ and KMnO_4_, diluted with ultrapure water, and treated with 30% H_2_O_2_. The resulting product underwent centrifugation and was washed with 1:10 HCl aqueous solution and ultrapure water in succession to remove ion species. Finally, few-layer graphene oxide was separated through centrifugation at 4000 rpm, yielding a GO suspension with a concentration of 5 mg/mL. Ultrapure water was acquired from our lab’s ultrapure water machine (MERCK, Burlington, MA, USA), exhibiting exceptional electrical resistivity of 18.2 MΩ·cm^−1^. CuPC was obtained from Aladdin Biological Technology Co., Ltd. (Shanghai, China). All materials were used as received without further purification.

To synthesize the CuPC@GO composite membrane, a preparation solution was obtained by mixing a 1 mM CuPC aqueous dispersion system and a 5 mg/mL GO suspension in equal volumes. Subsequently, ultra-pure water of the same volume as the preparation liquid was added for 1 h ultrasonic treatment to produce a thorough mixed CuPC@GO suspension. The resulting suspension was then divided into equal volume droplets, which were deposited onto release paper for self-assembly and drying for 12 h. This process ultimately yielded the CuPC@GO composite membrane.

During the assembly process, the flexible indium tin oxide (ITO)-coated polyethylene terephthalate (PET) substrate first was subjected to a sequential cleaning process involving acetone and isopropyl alcohol for a duration of 20 min each. It was then rinsed with ultrapure water and subsequently dried using N_2_ gas. Trimming the CuPC@GO membrane into 1 × 1 cm^2^ squares for further device assembly. Finally, we encapsulated the CuPC@GO piezoelectric pressure sensor (Figure 1a) with appropriately sized PET and polyimide (PI) films according to the PET/ITO/CuPC@GO/Au/PI structure, which ensures good contact between the electrodes and the CuPC@GO, and prevents damage from the external environment, where the CuPC@GO composite membrane serves as the pressure-sensing layer.

### 3.2. Characterization of CuPC@GO Membrane

The morphology of the CuPC@GO composite film was observed using a scanning electron microscope (SEM, ZEISS Gemini 300, Oberkochen, Germany) at an accelerating voltage of 3 kV, equipped with the energy dispersive spectrometer (EDS). The interlayer distances changes were studied using an X-ray diffractometer (XRD, Rigaku Ultimate IV, Tokyo, Japan). In order to elucidate the microstructure of the CuPC@GO membrane in greater detail, we used an X-ray photoelectron spectrometer (XPS, Thermo Fisher ESCALAB 250Xi, Winthrop, MA, USA) for surface and depth analysis of CuPC@GO composite films.

### 3.3. Piezoelectric Properties/Light Enhanced Piezoelectric Testing

The response speeds of the CuPC@GO piezoelectric pressure sensor were recorded using an HF2LI 50 MHz Lock-in Amplifier (Zurich Instruments, Zurich, Switzerland) at a sampling frequency of 1000,000 times per second, and response values were obtained by reducing the sampling frequency using the same instrument. The sensor unit is mounted on a heavy mechanical base in order to avoid false signals due to vibrations from mechanical components outside the test equipment. Pressure is applied by weights and solenoids and calibrated with commercial pressure transducers. The incident light sources, generated by several monochromatic lasers with adjustable power density, were carefully calibrated using photodiode power sensors (Thorlabs, Newton, NJ, USA; S120VC and PM100D). The illumination position and area were consistent for all measurements. It is worth noting that no light source other than the laser was present in the measurement environment. Moreover, the piezoelectric pressure sensor was tested under controlled conditions of room temperature (25 °C), 20% humidity, and atmospheric pressure.

### 3.4. Theoretical Calculations

Density functional theory (DFT) calculations were performed as implemented in the Vienna ab initio Simulation Package (VASP) code [28]. The projector augmented wave (PAW) pseudopotentials were applied [29]. The generalized gradient approximation (GGA) of the Perdew–Burke–Ernzerhof (PBE) was used to treat the exchange-correlation interaction between electrons [30]. The van der Waals interactions were introduced in the calculations, and they were described by a correction through Grimme’s zero-damping D-3 method [31]. The electron wave function was expanded by a plane-wave basis up to 500 eV. The Brillouin zones were sampled by k-point grids of 2 × 2 × 1 [32]. 

To avoid possible interlayer interaction, an empty space of 20 Å was used along the non-periodic direction. In structural relaxations, all ionic positions and the shape and volume of the supercell were allowed to relax until the residual force on each atom was less than 0.01 eV/Å. A dipole correction along the z-direction is considered in all calculations to correct the artificial electric polarization introduced by the periodic boundary condition and to balance the vacuum level differences on the different sides of the CuPC@Gra bilayers [33]. The Bader charge analysis was utilized to determine the electron charge transfer occurring between CuPC and graphene [34]. 

To construct the excited electronic state, we let the higher level (the energy level above the VBM) be pre-occupied by the electron from the VBM state, leaving the VBM level unoccupied. With the occupation of these electronic states fixed, CuPC@Gra will exhibit varying charge distributions [35]. 

## 4. Conclusions

In conclusion, we present a flexible self-powered piezoelectric sensor based on a composite membrane of CuPC and GO, the pressure-sensing performance of which was highly enhanced by external light illumination. Under light illumination, the sensor exhibited a larger output voltage (381.17 mV, 50 kPa), and a higher sensitivity (116.80 mV/kPa) within the range of *P* < 5 kPa, which was two times and three times that obtained from the unilluminated sensor. Moreover, the external light illumination improved the response time to 38.04 µs, demonstrating an ultra-fast response, while maintaining 95% performance stability after 2000 loading/unloading cycles. The underlying mechanism is attributed to the light illumination promoting the electron emission of the CuPC molecule, which can enable more electrons to be transferred from CuPC to graphene, resulting in the large polarization and the enhanced pressure-sensing performance of the sensor. The response voltage to external conditions, including pressure and the light illumination of the flexible piezoelectric sensor, is self-generated, eliminating the need for an external power source. This self-powered sensing capability significantly simplifies device operation and the construction of measurement circuits. 

The findings provide a straightforward fabrication technique so that the photoexcitable material, such as CuPC, can be simply combined with GO to form a membrane through self-assembly. It can serve as the sensing layer in a piezoelectric sensor, wherein the piezoelectric effect is enhanced through optical stimulation, resulting in a highly efficient flexible piezoelectric sensor. This mechanism permits high tunability of such sensors in a contactless manner. The straightforward fabrication process for the sensing layer materials and the ensuing light-enhanced functionality of self-powered flexible piezoelectric sensors presented in this study have the potential to yield unprecedented cost-effectiveness and provide opportunities for continuous health monitoring, clinical medicine, human identification, and a wide array of new smart electronic devices and systems.

## Data Availability

The data presented in this study are available in the article.
